# Functional connectivity in EEG: a multiclass classification approach for disorders of consciousness

**DOI:** 10.3389/fnins.2025.1550581

**Published:** 2025-03-28

**Authors:** Sreelakshmi Raveendran, Kala S, Ramakrishnan A G, Raghavendra Kenchaiah, Jayakrushna Sahoo, Santhos Kumar, Farsana M K, Ravindranadh Chowdary Mundlamuri, Sonia Bansal, Binu V S, Subasree R

**Affiliations:** ^1^Department of Electronics and Communication Engineering, Indian Institute of Information Technology Kottayam, Kerala, India; ^2^Department of Electrical Engineering, Indian Institute of Science, Bangalore, India; ^3^Centre for Neuroscience, Indian Institute of Science, Bangalore, India; ^4^Department of Neurology, NIMHANS, Bangalore, India; ^5^Department of Computer Science and Engineering, Indian Institute of Information Technology Kottayam, Kerala, India; ^6^Department of Neuroanaesthesia and Neurocritical Care, NIMHANS, Bangalore, India; ^7^Department of Biostatistics, NIMHANS, Bangalore, India

**Keywords:** functional connectivity, sliding window correlation, amplitude envelope correlation, weighted phase lag index, disorders of consciousness, artificial neural networks, LSTM, hybrid CNN-LSTM

## Abstract

Characterizing functional connectivity (FC) in the human brain is crucial for understanding and supporting clinical decision making in disorders of consciousness. This study investigates FC using sliding window correlation (SWC) analysis of electroencephalogram (EEG) applied to three connectivity measures: phase-lag index (PLI) and weighted phase-lag index (wPLI), which quantify phase synchronization, and amplitude envelope correlation (AEC), which captures amplitude-based coactivation patterns between pairs of channels. SWC analysis is performed across the five canonical frequency bands (delta, theta, alpha, beta, gamma) of EEG data from four distinct groups: coma, unresponsive wakefulness syndrome, minimally conscious state, and healthy controls. The extracted SWC metrics, mean, reflecting the stability of connectivity, and standard deviation, indicating variability, are analyzed to discern FC differences at the group level. Multiclass classification is attempted using various models of artificial neural networks that include different multilayer perceptrons (MLP), recurrent neural networks, long-short-term memory networks, gated recurrent units, and a hybrid CNN-LSTM model that combines convolutional neural networks (CNN) and long-short-term memory network to validate the discriminative power of these FC features. The results show that MLP model 2 achieves a classification accuracy of 96.3% using AEC features obtained with a window length of 16s, highlighting the effectiveness of AEC. An evaluation of the model performance for different window sizes (16 to 20 s) shows that MLP model 2 consistently achieves high accuracy, ranging from 95.5% to 96.3%, using AEC features. When AEC and wPLI features are combined, the maximum accuracy increases to 96.9% for MLP model 2 and 96.7% for MLP model 3, with a window size of 17 seconds in both cases.

## 1 Introduction

Functional connectivity (FC) has emerged as a transformative concept in neuroscience, offering a deeper understanding of brain function. FC allows researchers to explore brain's adaptability to external stimuli, cognitive challenges, and internal states (Sastry and Banerjee, 2024; Anusha et al., 2025). FC provides a more detailed picture of neural interactions by examining the synchronization patterns between different brain regions. This approach improves our understanding of the functional organization and flexibility of the brain. The ability to capture changes in FC is important for understanding complex cognitive processes and the brain's responses to ever-changing demands (Sastry and Banerjee, 2024).

Technologies such as functional magnetic resonance imaging (fMRI) (Mäki-Marttunen, 2014), electroencephalography (EEG) (Duclos et al., 2021; Dey et al., 2023), magnetoencephalography (MEG) (Jin et al., 2023), and functional near-infrared spectroscopy (fNIRS) (Tang et al., 2021) have been instrumental in advancing the study of functional connectivity. These neuroimaging modalities, supported by advancements in sensor resolution and computational techniques, enable the recording and analysis of FC patterns with high precision. Each modality offers unique advantages: fMRI detects blood oxygenation changes at a high spatial resolution, enabling whole-brain connectivity mapping (Mäki-Marttunen, 2014). EEG delivers exceptional temporal resolution by capturing electrical activity from the scalp, tracking rapid neural changes (Duclos et al., 2021). MEG measures magnetic fields produced by neural activity (Jin et al., 2023) at good spatial and temporal precision, while fNIRS uses near-infrared light to monitor cortical blood flow, offering portability and robustness to movement artifacts (Tang et al., 2021). These modalities have been particularly effective in studying resting-state networks, such as the default mode network and the salience network (Matsui and Yamashita, 2023). The spontaneous fluctuations within these networks illuminate baseline cognitive functions and introspective processes. A commonly employed method to analyze FC is the sliding window approach, which calculates connectivity metrics within overlapping time windows (Mokhtari et al., 2019). This technique allows researchers to track fluctuations in connectivity, providing insights into brain networks over time. The sliding window approach segments time series data into consecutive, overlapping windows of a specified length. Within each window, connectivity measures, such as the Pearson correlation coefficient, are calculated between pairs of brain regions, or electrode pairs (Saideepthi et al., 2023). Shifting the window across the data set can generate a time-resolved representation of connectivity. This method helps identify transient connectivity states and understand the brain's adaptability to various cognitive tasks and resting conditions.

Functional connectivity has facilitated significant advancements in medical research, particularly in the diagnosis and monitoring of neurological disorders such as Alzheimer's disease (Matsui and Yamashita, 2023), epilepsy (Qin et al., 2024), and stroke (Wu et al., 2024). Alterations in FC patterns have been associated with cognitive impairments in Alzheimer's patients, providing biomarkers of disease progression (Arbabyazd et al., 2023). In epilepsy, FC has offered critical insight into seizure mechanisms, which has helped to develop targeted therapies (Li et al., 2024b). It has also deepened our understanding of altered states of consciousness, such as those induced by anesthesia (Miao et al., 2023), psychedelics (Soares et al., 2024), and sleep (Xu et al., 2024). Under anesthesia, studies using techniques such as sliding window correlation (SWC) have revealed a reduction in the repertoire of FC states, reflecting a loss of the characteristic of dynamic flexibility of the brain of consciousness (Miao et al., 2023). However, psychedelics such as psilocybin expand the repertoire of FC and increase entropy, indicating heightened consciousness (Soares et al., 2024). Similarly, sleep studies reveal that deep sleep narrows the FC repertoire, reducing variability and diminishing the capacity for consciousness (Xu et al., 2024).

Beyond altered states, FC has proven valuable in neuropsychiatry (Matsui and Yamashita, 2023) by identifying disrupted connectivity patterns in conditions such as schizophrenia, depression, and anxiety. In schizophrenia, disruptions in FC correlate with cognitive deficits (Cattarinussi et al., 2023), while in depression, altered patterns are linked to symptom severity and treatment response, enabling personalized therapy. FC also elucidates developmental trajectories, showing increased stability and adaptability during maturation and reduced flexibility in aging and neurodegeneration, which reflects declining neural plasticity. Advances in computational techniques, including sliding window correlation, hidden Markov models, and multivariate autoregressive models, have enhanced the precision of FC analyses (Hutchison et al., 2013).

Beyond medical applications, FC contributes to cognitive science by revealing network interactions in processes like memory, attention, and learning. This knowledge informs strategies to enhance cognitive performance and supports advancements in brain-computer interfaces (BCIs). BCIs leverage FC to decode brain activity, enabling communication for individuals with disabilities, controlling prosthetic devices, and enhancing immersive gaming experiences (Fallani and Bassett, 2019). FC has also been used successfully in EEG-based biometrics (Kumar et al., 2022). FC also informs neuroergonomics by optimizing task environments to align with human cognitive capabilities, reducing mental fatigue, and improving performance (Ismail and Karwowski, 2020). It sheds light on social interactions by analyzing synchronized FC patterns during communication, offering insights into the neural basis of empathy and social connectivity. FC has found transformative applications in understanding and managing disorders of consciousness (DOC). In these cases, FC differentiates between minimally conscious states (MCS) and unresponsive wakefulness syndrome (UWS), since reduced variability and connectivity integration are associated with lower levels of consciousness. Stronger connectivity patterns in MCS patients align with a relatively higher degree of awareness (Naro et al., 2018). Clinically, FC assists in prognosis by identifying recovery markers, such as restored corticothalamic connectivity, and reveals covert awareness in unresponsive patients, guiding personalized care and rehabilitation. While existing literature highlights the potential of FC in understanding and managing consciousness in DOC cohorts, significant ambiguity persists in effectively distinguishing between various consciousness states and healthy controls. This lack of clarity hampers the development of reliable diagnostic and prognostic tools, creating a need for advanced analytical approaches.

This work investigates the classification of different states of consciousness: coma, UWS, MCS, and healthy controls (HC) using functional connectivity features derived from resting state EEG data. By leveraging metrics such as wPLI and AEC, the study aims to analyze brain connectivity patterns to differentiate between these states. The performance analysis has been conducted for different window sizes (16 to 20 s with a step size of 1 s). The analysis incorporates sliding window correlation to explore the variability in connectivity, providing insights into how brain network characteristics vary across different levels of consciousness.

## 2 Materials and methods

### 2.1 Resting state EEG measurement

The resting state brain activity of the subjects recruited for the study is acquired using the EEG machine with Galileo software at NIMHANS, Banglore. The recording duration is chosen as 30 minutes. Electrodes are set according to the international 10/10 system, using a sampling rate of 256 Hz and ensuring that impedance remains below 5 kΩ. The EEG data is referenced to the left (A1) and right earlobes (A2). The dataset includes 16 patients from coma, 20 subjects from UWS, MCS, and healthy controls. EEG recordings are performed while participants are positioned supine with their eyes closed, and methods are implemented to reduce artifacts (Raveendran et al., 2023). Consultant neurologists have recruited and classified the patients into various consciousness categories based on established behavioral assessment methods of CRS-R (coma recovery scaled-revised) and GCS (Glasgow coma scale). Further details regarding patient recruitment, inclusion, and exclusion criteria, as well as the signal acquisition protocol, are available in Raveendran et al. (2024).

### 2.2 Data preprocessing

The resting-state EEG signals are visually inspected by expert EEG technicians to detect bad channels, artifact-affected epochs, and recorded annotations. Subsequently, appropriate channels are selected and average referencing is applied to the signal. The preprocessing steps applied include trimming the first and last two minutes to remove initial setup artifacts and ending noise followed by adding a standard 10-20 montage to ensure correct spatial mapping of electrodes. A band-pass filter (1–48 Hz) is applied to remove low-frequency drifts and high-frequency muscle noise, followed by a notch filter at 50 Hz to eliminate power line interference. Bad segments were automatically rejected based on annotations. The five distinct EEG frequency bands were extracted from the preprocessed signals using a zero-phase, non-causal FIR bandpass filter. This filter was designed with a windowed time-domain approach (firwin), setting the lower and upper cut-off frequencies according to the respective EEG bands. A Hamming window was applied, featuring a passband ripple of 0.0194 and a stopband attenuation of 53 dB (Raveendran et al., 2024). To maintain the same number of subjects in each group, 15 patients are selected for analysis. The selection of the 15 subjects was based on two key criteria. First, only subjects with available follow-up data were included to ensure longitudinal consistency in the analysis. Second, subjects with the longest EEG signal duration after preprocessing were chosen to maximize the number of extracted windows, thereby enhancing the reliability and robustness of the analysis. A 20-s sliding window with 50% overlap is applied to each record to generate analysis segments. Windows are extracted from each channel and frequency band. 95 windows are extracted from each signal of each patient, taking it to a total of 475 windows from each subject. The total number of windows in each group is 7,125; making a data sample set of 28,500 for all four classes. For analysis, the FC features wPLI and AEC are extracted from these 28,500 windows. The study also explores the impact of different window lengths (ranging from 16 to 20 s) on data segmentation and the overall dataset size. A 16 s window size results in 36,000 data samples, with each group contributing 9,000 samples.

## 3 Methodology

### 3.1 Feature extraction

#### 3.1.1 Phase lag index (PLI)

The phase lag index (PLI) is a metric widely used in neuroscience for quantifying functional connectivity between two signals based on their phase relationships. PLI focuses on the consistency of non-zero phase lags, making it robust against the effects of common-source artifacts. This robustness is advantageous in studying brain connectivity, where eliminating spurious correlations is critical for uncovering genuine interactions. PLI is equal to 0 when there is no consistent phase lag between signals, indicating that the phase differences are symmetrically distributed around zero, which can occur in cases of perfect synchrony or random phase differences. When the phase difference is consistently ±π (exactly 180 degrees out of phase), then the rate of change or slope of phase difference with respect to frequency is zero. Hence, PLI is zero.

The phase, which encodes information about the temporal coordination of oscillatory activity, is extracted from the analytic representation of a real-valued signal *x*(*t*) using its Hilbert transform *H*(*x*(*t*)). The analytic signal *z*(*t*) of *x*(*t*) is expressed as:


z(t)=x(t)+iH(x(t))

The amplitude envelope *A*_*x*_(*t*) is the magnitude of the analytic signal:


$$A_{x}\left(t\right)=|z\left(t\right)|=\sqrt{x{\left(t\right)}^{2}+H{\left(x\left(t\right)\right)}^{2}}$$

and its instantaneous phase is given by:


$$\phi_{x}\left(t\right)=\tan^{-1}\left(\frac{\text{Im}\left(z\left(t\right)\right)}{\text{Re}\left(z\left(t\right)\right)}\right)$$

For two signals *x*(*t*) and *y*(*t*), the instantaneous phase difference is:


$$\Delta \phi \left(t\right)=\phi_{x}\left(t\right)-\phi_{y}\left(t\right)$$

The PLI is derived from these phase differences and emphasizes the asymmetry of consistent phase lags (Stam et al., 2007). It is defined as:


PLI=|𝔼[sgn(sin(Δϕ(t))]|

Here, 𝔼[·] denotes the expectation (or average) over time, sgn(*sin*(Δϕ(*t*))) is the sign of the sine of the phase difference. The PLI quantifies the consistency of directional phase relationships by focusing on whether one signal consistently leads or lags the other. The normalized range between 0 and 1 makes it well-suited for comparing connectivity patterns across different datasets and experimental conditions, establishing it as a robust measure for studying functional brain networks.

#### 3.1.2 Weighted phase lag index (wPLI)

The weighted phase lag index (wPLI) extends the PLI by incorporating the magnitude of phase lag asymmetry, providing a more sensitive measure of functional connectivity. Like PLI, the wPLI focuses on non-zero phase lags. However, by weighting the contribution of phase differences based on the magnitude of their imaginary components, the wPLI enhances sensitivity to true underlying interactions, particularly in noisy data (Vinck et al., 2011). With the instantaneous phases of the signals derived from their analytic representations, the wPLI is calculated as:


$$\text{wPLI}=\frac{\left|𝔼\left[\text{Im}\left(\Delta \phi \left(t\right)\right)\cdot \text{sgn}\left(\text{Im}\left(\Delta \phi \left(t\right)\right)\right)\right]\right|}{𝔼\left[\left|\text{Im}\left(\Delta \phi \left(t\right)\right)\right|\right]}$$

where, Im(Δϕ(*t*)) is the imaginary part of the phase difference, and sgn(Im(Δϕ(*t*))) is the sign function (Vinck et al., 2011). The numerator emphasizes the magnitude of phase lag asymmetry, while the denominator normalizes the result to bound wPLI values between 0 and 1. A wPLI value close to 0 indicates no consistent phase lag, while values close to 1 indicate strong phase synchronization with consistent lag. The bounded range of wPLI facilitates comparisons across datasets and conditions, making it a reliable measure for studying functional brain networks.

Both PLI and wPLI quantify the consistency of phase differences between signals; however they differ in their weighting mechanisms. PLI is based on the sign of the imaginary component of the cross-spectrum, measuring whether phase differences are systematically positive or negative. PLI equally weights all samples, including those where the phase difference is close to 0 or ±π which can introduce noise and reduce sensitivity. wPLI addresses this limitation by weighting the contribution of each sample by the magnitude of the imaginary component of the cross-spectrum. Consequently, wPLI provides a more reliable measure of true brain interactions by emphasizing phase differences with stronger imaginary components, thereby improving robustness against common-source artifacts.

#### 3.1.3 Amplitude envelope correlation (AEC)

While phase-based measures like wPLI assess temporal coordination in oscillatory activity, amplitude-based methods such as amplitude envelope correlation (AEC) provide complementary insights by focusing on the co-variation of signal intensities (Bruns et al., 2000). AEC specifically quantifies the linear relationship between the amplitude envelopes of two signals over time, capturing how their intensities fluctuate in tandem.

The amplitude envelopes required for AEC are obtained from the same analytic signal derived using the Hilbert transform, as described earlier. Given the amplitude envelopes *A*_*x*_(*t*) and *A*_*y*_(*t*) of two signals *x*(*t*) and *y*(*t*), AEC is calculated as the Pearson correlation coefficient between these envelopes over a specified time interval *T*:


$$\text{AEC}=\frac{\sum_{t=1}^{T}\left(A_{x}\left(t\right)-{Ā}_{x}\right)\left(A_{y}\left(t\right)-{Ā}_{y}\right)}{\sqrt{\sum_{t=1}^{T}{\left(A_{x}\left(t\right)-{Ā}_{x}\right)}^{2}\sum_{t=1}^{T}{\left(A_{y}\left(t\right)-{Ā}_{y}\right)}^{2}}}$$

where Ā_*x*_ and Ā_*y*_ are the respective mean values of *A*_*x*_(*t*) and *A*_*y*_(*t*) over *T*.

AEC captures slow fluctuations in signal power, often associated with large-scale brain network activity, such as in studies of the resting state. AEC focuses on the comodulation of signal envelopes, making it a functionally relevant metric. Although AEC does not inherently eliminate volume conduction due to the presence of zero-lag correlations, it provides valuable insights into frequency-dependent connectivity beyond pure phase synchrony.

## 4 Sliding window correlation (SWC)

Sliding window correlation is a widely used method for examining the time-varying relationships between two signals or datasets. Traditional static correlation assumes that the relationship between signals remains constant over time. However, these relationships are often nonstationary and evolve in real-world scenarios, such as neural activity, physiological signals, and financial systems. Sliding window correlation has emerged as a prominent method for capturing these time-varying dependencies.

SWC involves partitioning the signals into smaller temporal windows, calculating the correlation within each window, and observing how the correlation evolves as the window “slides” across the signals. This method has been instrumental in understanding functional connectivity, particularly in neuroscience, where it provides insights into the temporal fluctuations of brain network interactions.

SWC builds on the standard Pearson correlation coefficient, a widely used metric for measuring the linear relationship between two-time series. The Pearson correlation coefficient is defined as:


$$r=\frac{\sum_{t=1}^{T}\left(X_{t}-\bar{X}\right)\left(Y_{t}-Ȳ\right)}{\sqrt{\sum_{t=1}^{T}{\left(X_{t}-\bar{X}\right)}^{2}\sum_{t=1}^{T}{\left(Y_{t}-Ȳ\right)}^{2}}}$$

where; *X*_*t*_, *Y*_*t*_ are the values of the two signals at time t and $\bar{X}$, Ȳ are the mean values of *X*_*t*_ and *Y*_*t*_ over the time interval T. A 20-second sliding window with 50% overlap is applied to obtain the windows of the input EEG signal. Within each window k, the Pearson correlation is computed as:


$$r_{k}=\frac{\sum_{t=t_{k}}^{t_{k}+W-1}\left(X_{t}-{\bar{X}}_{k}\right)\left(Y_{t}-{Ȳ}_{k}\right)}{\sqrt{\sum_{t=t_{k}}^{t_{k}+W-1}{\left(X_{t}-{\bar{X}}_{k}\right)}^{2}\sum_{t=t_{k}}^{t_{k}+W-1}{\left(Y_{t}-{Ȳ}_{k}\right)}^{2}}}$$

**Figure 1:**
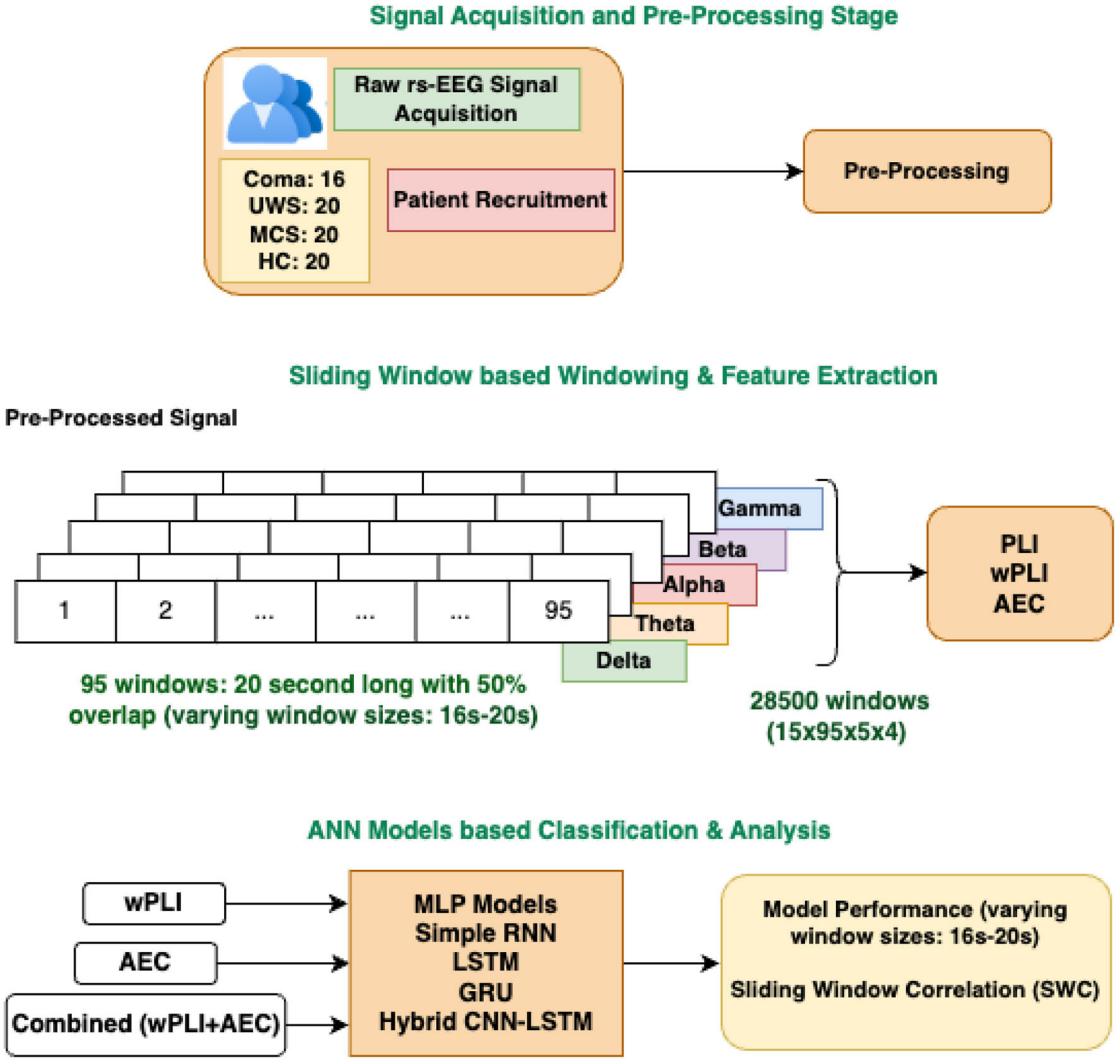
Graphical depiction of the proposed methodology: FC-based multiclass classification of DOC patients.

Here *t*_*k*_ is the starting time of the k-th window, $\bar{X_{k}}$ and $\bar{Y_{k}}$ are the mean values of *X*_*t*_ and *Y*_*t*_ within the k-th window. The window is then shifted by a step size S and the process is repeated until the entire signal is analyzed. The result is a time series of correlation coefficients *r*_*k*_ representing the temporal evolution of the relationship between X and Y. The proposed methodology is graphically depicted in Figure 1.

## 5 Choice of ANN models for multiclass classification

The proposed work employs several advanced artificial neural network (ANN) architectures to classify states of consciousness–coma, UWS, MCS, and healthy subjects–using functional connectivity features, namely PLI, wPLI and AEC. The architectures include three variations of multilayer perceptron (MLP), simple recurrent neural network (RNN), long short-term memory network (LSTM), gated recurrent units (GRUs), and a hybrid CNN-LSTM model, each tailored to optimize the learning process for different data representations.

The preprocessed signal has 19 channels: F3, F4, C3, C4, F7, F8, P3, P4, T7, T8, P7, P8, O1, O2, Fp1, Fp2, Fz, Pz, and Cz. Each channel is divided into five frequency bands (delta, theta, alpha, beta, and gamma). Thus each connectivity feature gives rise to a symmetric feature matrix of size 19 × 19 for each frequency band. Due to its symmetry, only the upper triangular elements, excluding the diagonal, are used for classification. Hence, the number of unique elements is 171 per frequency band. With 5 frequency bands, the total feature vector for each window contains 5 × 171 = 855 values. For 20 s window size, each subject generates 95 windows, resulting in a feature matrix of shape (95,855) per subject. With 15 subjects per group, the total number of samples per group is 15 × 95 = 1,425. Considering all four groups, the dataset comprises 4 × 1,425 = 5,700 samples, each represented by a feature vector of dimension 855. After reshaping the data into a 2D array, the final input has a shape of (5,700,855). For a window size of 19 s, each subject produces 103 windows, leading to a feature matrix of shape (103, 855) per subject. Given 15 subjects per group, the total number of samples per group is 1,545 (15 × 103). Considering four groups, the input comprises 6,180 samples (4 × 1,545). When the window size is 18 s, each subject generates 109 windows, resulting in a final feature matrix of shape (6,540,855) for all the groups together (109 X 15 4 = 6,540). For 17 s window size, each subject produces 115 windows, resulting in a dataset of 6,900 feature vectors. With a window size of 16 s, each subject generates 120 windows, finally resulting in 7,200 feature vectors.

### 5.1 MLP models

The MLP model 1 starts with an input layer that accepts flattened input data of shape (X_train.shape[1]), where the number of neurons corresponds to the total features in the dataset. This ensures compatibility with fully connected layers and simplifies computations. It employs a dense hidden layer with 128 neurons and ReLU activation, followed by dropout regularization with a rate of 0.5 to prevent overfitting. A second hidden layer contains 64 neurons, also with ReLU activation, and applies a dropout of 0.3 for additional regularization. The output layer has four neurons (one for each class), with a softmax activation function to produce class probabilities. The optimizer used is Adam with default hyperparameters, and the loss function is categorical crossentropy, suitable for multiclass classification. Training involves 50 epochs with a batch size of 32, using 10% of the training data as a validation split.

The MLP model 2 builds upon the first model and starts with an input layer identical to model 1, where the number of neurons matches the feature size (X_train.shape[1]). It incorporates a dense hidden layer with 256 neurons and ReLU activation, followed by batch normalization to stabilize and accelerate training. Dropout is applied with a rate of 0.4. A second hidden layer with 128 neurons employs ReLU activation, batch normalization, and dropout at 0.4. A third hidden layer contains 64 neurons with ReLU activation and dropout at 0.3. The second and third layers introduce kernel regularization with L2 penalty (0.001) to further combat overfitting. Like model 1, the output layer uses softmax activation with four units, optimized by Adam with a learning rate of 0.001. Training parameters are identical to those of model 1.

MLP model 3 resembles model 2 but maintains an identical input layer, where the number of neurons is X_train.shape[1]. It includes two hidden layers with 256 and 128 neurons, respectively, both employing ReLU activation, batch normalization, dropout (0.4), and L2 regularization (0.001). The output layer is configured identically to the first two models, and the optimizer is Adam. Early stopping and learning rate reduction callbacks are used to halt training when validation performance stops improving, ensuring optimal resource usage.

### 5.2 Sequential models: simple RNN, LSTM, and GRU

The simple RNN model processes data as sequences, requiring input to be reshaped into (time_steps, features). The first recurrent layer employs 128 simple RNN units with tanh activation and return_sequences = True to retain outputs for the next layer. Dropout regularization is applied with a rate of 0.4. A second recurrent layer with 64 simple RNN units has return_sequences = False, followed by dropout at 0.3. Batch normalization is applied after each layer to stabilize gradients. Dense layers are used post-RNN, starting with a layer of 32 units, ReLU activation, and dropout of 0.2, culminating in an output layer with four softmax-activated neurons. Adam optimizer is used with a learning rate of 0.0005, and categorical cross-entropy is the loss function. The performance evaluation metrics include accuracy, precision, recall, and F1-score.

The LSTM model leverages the long-term memory capabilities of LSTM layers. The first LSTM layer has 128 units, return_sequences = True, and applies dropout at 0.4 to reduce overfitting. The second LSTM layer, with 64 units and return_sequences = False, uses dropout at 0.3. Batch normalization is applied after each layer. Post-LSTM dense layers include a 32-unit layer with ReLU activation and dropout at 0.2, followed by the output layer with four softmax-activated neurons. Adam optimizer with a learning rate of 0.0005 is used, along with early stopping and learning rate reduction callbacks.

The GRU model is structured similarly to the LSTM model but replaces LSTM layers with GRU layers, which are computationally more efficient while retaining the ability to model sequential dependencies. The first GRU layer has 128 units with return_sequences = True and dropout at 0.4. The second GRU layer, with 64 units and return_sequences = False, uses dropout at 0.3. Batch normalization follows each GRU layer. The dense layers include a 32-unit layer with ReLU activation and dropout at 0.2, followed by the output layer with four softmax-activated neurons. Like the LSTM model, this configuration uses Adam optimizer (learning rate 0.0005), categorical cross-entropy, and early stopping to ensure optimal training performance.

A hybrid CNN-LSTM model integrates convolutional and recurrent architectures for effective sequential learning. The input data is structured as (time_steps, num_features), capturing temporal dependencies. The model begins with two Conv1D layers (64 and 128 filters) using ReLU activation, followed by MaxPooling1D layers to downsample the feature maps. Batch normalization is applied after each convolutional layer to stabilize training. The extracted spatial features are then passed through two LSTM layers (64 and 32 units) with tanh activation, modeling sequential dependencies. Dropout (0.3 and 0.2) is used in LSTM layers to prevent overfitting. A fully connected dense layer with 32 neurons (ReLU activation) and dropout (0.2) is followed by a softmax-activated output layer for multi-class classification. The model is trained using categorical cross-entropy loss and optimized with the Adam optimizer (learning rate 0.0005). It is trained with a batch size of 32 for 50 epochs, incorporating early stopping and a learning rate reduction scheduler to enhance convergence and performance. Table 1 describes the network configurations and the model details.

**Table 1:** Description of the implemented ANN models for classification.

**Model**	**Architecture (layers)**	**Units summary**	**Activation functions**	**Optimizer (LR)**	**Batch size**	**Epochs**
MLP model 1	Dense → Dense → Dense	128 → 64 → 4	ReLU, Softmax	Adam (Default)	32	50
MLP model 2	Dense → Dense → Dense	256 → 128 → 64 → 4	ReLU, Softmax	Adam (0.001)	32	100
MLP model 3	Dense → Dense → Dense	256 → 128 → 64 → 4	ReLU, Softmax	Adam (0.001)	32	60
Simple RNN	RNN → RNN → Dense	128 → 64 → 32 → 4	tanh, ReLU, Softmax	Adam (0.0005)	32	50
LSTM	LSTM → LSTM → Dense	128 → 64 → 32 → 4	tanh, ReLU, Softmax	Adam (0.0005)	32	50
GRU	GRU → GRU → Dense	128 → 64 → 32 → 4	tanh, ReLU, Softmax	Adam (0.0005)	32	50
Hybrid CNN-LSTM	Conv1D → Conv1D → LSTM → LSTM → Dense	64 → 128 → 64 → 32 → 4	ReLU, ReLU, tanh, ReLU, Softmax	Adam (0.0005)	32	50

Training and Evaluation: All models are trained using standardized inputs to ensure consistency across features. Labels are one-hot encoded to suit the categorical classification task. During training, 10% of the data is reserved for validation to monitor overfitting. Metrics such as accuracy, weighted precision, recall, and F1-score provide a holistic evaluation of model performance, especially for imbalanced class distributions.

## 6 Results

Table 2 compares the six models using the performance metrics of accuracy, precision, recall, and F1-score. The metrics used compare the effectiveness of various artificial neural network models–3 MLP models, simple RNN, LSTM, and GRU–for classifying states of consciousness based on the functional connectivity features of phase lag index, weighted phase lag index, and amplitude envelope correlation. Table 2 demonstrates the distinct trends influenced by the choice of features and model architectures in leveraging the FC data to classify states of consciousness into coma, UWS, MCS, and healthy subjects. The analysis has been conducted using different window sizes to examine the impact of window size on model performance. The applied sizes of the window are 16, 17, 18, 19, and 20 s, with a 50% overlap maintained to capture variability.

**Table 2:** Performance comparison of different ANN models for multiclass classification with 20s window length and 80/20 train-test split (coma vs. UWS vs. MCS vs. HC) using three different features and a combination of two of them.

**Performance metrics**	**Feature**	**MLP model 1**	**MLP model 2**	**MLP model 3**	**Simple RNN**	**LSTM**	**GRU**	**Hybrid CNN-LSTM**
Accuracy	PLI	0.548	0.622	0.618	0.52	0.533	0.529	0.571
	Precision	0.553	0.626	0.619	0.529	0.543	0.559	0.572
	Recall	0.548	0.622	0.618	0.52	0.533	0.529	0.571
	F1-score	0.550	0.624	0.619	0.524	0.537	0.544	0.571
Accuracy	wPLI	0.772	0.532	0.515	0.668	0.638	0.713	0.48
	Precision	0.773	0.613	0.706	0.671	0.646	0.718	0.55
	Recall	0.772	0.532	0.515	0.668	0.638	0.713	0.48
	F1-score	0.773	0.570	0.596	0.669	0.642	0.716	0.513
Accuracy	AEC	0.879	0.957	0.941	0.905	0.906	0.903	0.927
	Precision	0.879	0.958	0.942	0.907	0.908	0.904	0.928
	Recall	0.879	0.957	0.941	0.905	0.906	0.903	0.927
	F1-score	0.879	0.958	0.942	0.906	0.907	0.903	0.928
Accuracy	wPLI & AEC	0.874	0.951	0.948	0.902	0.918	0.921	0.925
	Precision	0.876	0.952	0.948	0.904	0.918	0.921	0.925
	Recall	0.874	0.951	0.948	0.902	0.918	0.921	0.925
	F1-score	0.875	0.951	0.948	0.903	0.918	0.921	0.925

### 6.1 Performance analysis on PLI features

For the PLI feature, MLP model 2 exhibits the highest performance across all metrics, with an accuracy of 0.622, precision of 0.626, recall of 0.622, and an F1-score of 0.624. These results demonstrate that MLP model 2 achieves a slightly better balance between precision and recall, leading to the highest F1-score among all the evaluated models. MLP models 1 and 3 follow closely in terms of performance, with MLP model 3 showing slightly better results than MLP model 1. The RNN-based models perform poorer. The simple RNN model achieves an accuracy of 0.52, precision of 0.529, recall of 0.52, and an F1-score of 0.524. Among the RNN-based architectures, the GRU model marginally outperforms the simple RNN and LSTM models, with an F1-score of 0.544, reflecting a slightly better precision-to-recall balance. The LSTM model achieves slightly better than the simple RNN with accuracy, precision, recall, and F1-score values of 0.533, 0.543, 0.533, and 0.537, respectively.

Table 3 presents the accuracy of different models for PLI across varying window sizes (16–20 s) using an 80-20 fixed train-test split. The results showed that larger window sizes (19 and 20 s) lead to better model performance, particularly for MLP architectures. Among the models, MLP Model 2 consistently achieves higher accuracy, peaking at 0.622 for the 20 s window, followed closely by MLP Model 3 at 0.6183. Recurrent models (simple RNN, LSTM, and GRU) exhibit lower overall performance. Both LSTM and GRU models improve in performance as the window size increases, reaching higher accuracy at 20 s (0.5325 and 0.5292, respectively).

**Table 3:** Accuracy with PLI features for different window sizes with 80-20 fixed train-test split.

**Model**	**16 s**	**17 s**	**18 s**	**19 s**	**20 s**
MLP model 1	0.5376	0.5513	0.5547	0.5594	0.5479
MLP model 2	0.6156	0.5974	0.6128	0.6197	0.622
MLP model 3	0.6111	0.5958	0.607	0.6063	0.6183
Simple RNN	0.5165	0.5058	0.5083	0.5076	0.52
LSTM	0.4735	0.4722	0.4797	0.4688	0.5325
GRU	0.4874	0.4684	0.4622	0.4782	0.5292
Hybrid CNN-LSTM	0.5502	0.5585	0.5608	0.561	0.5705

Table 4 presents model performance results using group k-fold cross-validation with PLI as the feature extraction method. The results show that MLP-based models consistently outperform RNN-based models, with MLP model 2 achieving the highest accuracy and F1-score across all window sizes. An increase in window size leads to improved performance, particularly for MLP models, highlighting their ability to leverage longer temporal dependencies more effectively. Among the cross-validation folds, folds 10 and 8 produce better results, especially for MLP models 2 and 3, suggesting that these data splits are more favorable for efficiently training the models. In contrast, recurrent models simple RNN, LSTM, and GRU exhibit greater variability in performance and underperform compared to MLP-based models.

**Table 4:** Best fold obtained using group k-fold cross validation and the performance matrix for each model using PLI.

**16 s**			**17 s**		**18 s**		**19 s**		**20 s**	
MLP model 1 CV: 10	Accuracy	0.5545	CV: 5	0.5633	CV: 8	0.5646	CV: 8	0.5727	CV: 8	0.5779
	Precision	0.5608		0.5653		0.567		0.5775		0.5822
	Recall	0.5545		0.5633		0.5646		0.5727		0.5779
	F1-score	0.5576		0.5642		0.5658		0.5751		0.58
MLP model 2 CV: 10	Accuracy	0.6151	CV: 8	0.6261	CV: 10	0.6258	CV: 8	0.6339	CV: 10	0.64
	Precision	0.616		0.627		0.6263		0.6347		0.6408
	Recall	0.6151		0.6261		0.6258		0.6339		0.64
	F1-score	0.6474		0.6596		0.6582		0.6665		0.6730
MLP model 3 CV: 10	Accuracy	0.6145	CV: 8	0.6264	CV: 10	0.6283	CV: 8	0.6339	CV: 8	0.6418
	Precision	0.6152		0.6275		0.6289		0.6345		0.6425
	Recall	0.6145		0.6264		0.6283		0.6339		0.6418
	F1-score	0.6145		0.6271		0.6299		0.6342		0.6431
Simple RNN CV: 10	Accuracy	0.5218	CV: 10	0.5238	CV: 8	0.5275	CV: 10	0.5376	CV: 8	0.5365
	Precision	0.5271		0.5296		0.5347		0.5449		0.5462
	Recall	0.5218		0.5238		0.5275		0.5376		0.5365
	F1-score	0.4819		0.4799		0.4850		0.4970		0.4933
LSTM CV: 5	Accuracy	0.4807	CV: 10	0.491	CV: 5	0.4861	CV: 8	0.4914	CV: 8	0.4866
	Precision	0.4869		0.4955		0.4912		0.4984		0.4892
	Recall	0.4807		0.491		0.4861		0.4914		0.4866
	F1-score	0.4641		0.4774		0.4688		0.4728		0.4642
GRU CV: 10	Accuracy	0.4996	CV: 10	0.4953	CV: 5	0.5	CV: 8	0.5076	CV: 5	0.502
	Precision	0.5065		0.4997		0.5058		0.5111		0.5059
	Recall	0.4996		0.4953		0.5		0.5076		0.502
	F1-score	0.5127		0.4996		0.5099		0.5176		0.5118
Hybrid CNN-LSTM CV: 10	Accuracy	0.562	CV: 8	0.5709	CV: 8	0.5711	CV: 10	0.5763	CV: 8	0.5822
	Precision	0.5629		0.5735		0.572		0.577		0.5832
	Recall	0.562		0.5709		0.5711		0.5763		0.5822
	F1-score	0.5624		0.5722		0.5715		0.5766		0.5827

Better results are obtained with MLP-based models, particularly models 2 and 3, using folds 8 and 10, and larger window sizes (19 and 20 s). The performance highlights that PLI-based features are better leveraged by MLP architectures rather than sequential models, potentially due to their ability to learn complex, high-dimensional representations more effectively.

### 6.2 Performance analysis on wPLI features

For the wPLI feature, which captures phase-based connectivity between brain regions, the performance shows significant variability across models. MLP model 1 emerges as the highest-performing model in terms of accuracy, achieving a value of 0.772. The high accuracy is supported by its precision of 0.773, which shows that it has a good ability to correctly identify positive predictions with relatively fewer false positives. Its recall value matches its accuracy at 0.772, suggesting that the model captures a large proportion of true positives. The F1-score of MLP model 1 is 0.773, reflecting a strong balance between precision and recall, which is critical in cases where the dataset may have an uneven class distribution or when both metrics are equally important for evaluation. This performance highlights that MLP model 1 can extract meaningful patterns from the wPLI feature set, leading to high classification success.

However, MLP models 2 and 3 significantly underperform. MLP model 2 achieves an accuracy of only 0.532. The corresponding precision (0.613), recall (0.532), and F1-score (0.570) further emphasize its struggle with generalization. MLP model 3 performs even worse, with an accuracy of 0.515. Both models exhibit poor adaptability to wPLI feature set, which may stem from their inability to effectively process the complex relationships embedded in the data.

The simple RNN model provides moderate performance, with an accuracy of 0.668. While it does not match the highest-performing MLP model 1 or GRU in accuracy, it surpasses MLP models 2 and 3 by a significant margin. The precision of 0.671 and recall of 0.668 are relatively balanced, and the F1-score of 0.669 confirms its consistent classification ability.

The LSTM network, a more advanced recurrent model, achieves an accuracy of 0.638. While this value is lower than that of simple RNN, its precision (0.646), recall (0.638), and F1-score (0.642) suggest that its performance is stable across metrics. However, LSTM does not perform as well as GRU, which surpasses it in all the metrics. This relative underperformance could be attributed to the additional complexity of LSTM, which may require more data and fine-tuning to optimize its performance on the wPLI feature set. LSTM's slightly lower accuracy compared to simple RNN highlights the trade-off between model complexity and effective learning, especially when working with limited datasets.

The GRU model stands out as a better performer in this comparison. With accuracy, precision, recall, and F1-score of 0.713, 0.718, 0.713, and 0.716, respectively, GRU demonstrates a well-rounded ability to classify instances in the wPLI feature set. Its F1-score is notable, reflecting a fine balance between precision and recall. This performance advantage is likely due to GRU's simpler architecture than LSTM, which allows it to efficiently capture the patterns without overfitting or requiring excessive computational resources. GRU's high precision also suggests that it minimizes false positives, making it a reliable choice for tasks where avoiding incorrect classifications is critical.

When comparing the models holistically, MLP model 1 achieves the highest accuracy, since it excels in extracting patterns. GRU, on the other hand, provides a more balanced performance across all metrics. This makes GRU the most effective model for the wPLI feature, since it leverages the dataset's unique temporal properties while maintaining strong generalization.

Accuracy across multiple deep learning models based on wPLI and different window sizes (16–20 s) using an 80-20 fixed train-test split is shown in Table 5. The results imply that increasing the window size improved accuracy for the GRU and LSTM models. However, MLP Model 1 has remained the most robust choice, showing stable and high accuracy across all window sizes.

**Table 5:** Accuracy with wPLI features for different window sizes with 80-20 fixed train-test split.

**Model**	**16 s**	**17 s**	**18 s**	**19 s**	**20 s**
MLP model 1	0.7535	0.7649	0.7694	0.7625	0.7719
MLP model 2	0.5086	0.5119	0.5772	0.5604	0.5319
MLP model 3	0.4811	0.5265	0.5751	0.4979	0.5151
Simple RNN	0.6428	0.6388	0.6977	0.6819	0.6679
LSTM	0.5729	0.6175	0.5538	0.6157	0.6377
GRU	0.61	0.6296	0.6072	0.6382	0.7132
Hybrid CNN-LSTM	0.4	0.445	0.465	0.475	0.48

The group k-fold cross-validation results for models trained using wPLI-based features demonstrate varying performances across different architectures and window sizes is shown in Table 6. MLP Model 1 consistently outperforms all other models, achieving the highest accuracy of 0.785 at 20 s using fold 10, indicating its robustness in capturing relevant patterns from the data. The simple RNN model also performed well, reaching an accuracy of 0.7171 at 20 s, with Fold 8 yielding the best results in multiple cases.

**Table 6:** Best fold obtained using group k-fold cross validation and the performance matrix for each model using wPLI.

**16 s**			**17 s**		**18 s**		**19 s**		**20 s**	
MLP model 1 CV: 10	Accuracy	0.7622	CV: 10	0.7713	CV: 8	0.7769	CV: 10	0.7827	CV: 10	0.785
	Precision	0.7631		0.7728		0.7781		0.7839		0.786
	Recall	0.7622		0.7713		0.7769		0.7827		0.785
	F1-score	0.7626		0.7720		0.7775		0.7833		0.7855
MLP model 2 CV: 8	Accuracy	0.5414	CV: 5	0.5509	CV: 10	0.5566	CV: 10	0.561	CV: 8	0.5798
	Precision	0.6639		0.643		0.6597		0.6652		0.6759
	Recall	0.5414		0.5509		0.5566		0.561		0.5798
	F1-score	0.5964		0.5934		0.6038		0.6087		0.6242
MLP model 3 CV: 10	Accuracy	0.5399	CV: 10	0.5661	CV: 10	0.5558	CV: 8	0.5672	CV: 5	0.5703
	Precision	0.6499		0.6651		0.6578		0.6643		0.6379
	Recall	0.5399		0.5661		0.5558		0.5672		0.5703
	F1-score	0.5898		0.6116		0.6025		0.6119		0.6022
Simple RNN CV: 8	Accuracy	0.6773	CV: 10	0.7015	CV: 8	0.7048	CV: 10	0.7106	CV: 8	0.7171
	Precision	0.6804		0.7042		0.7066		0.7123		0.7189
	Recall	0.6773		0.7015		0.7048		0.7106		0.7171
	F1-score	0.6788		0.7028		0.7057		0.7114		0.7180
LSTM CV: 8	Accuracy	0.6131	CV: 8	0.6293	CV: 10	0.6256	CV: 8	0.6378	CV:10	0.6317
	Precision	0.616		0.6326		0.6276		0.6416		0.6359
	Recall	0.6131		0.6293		0.6256		0.6378		0.6317
	F1-score	0.6145		0.6309		0.6266		0.6397		0.6338
GRU CV: 5	Accuracy	0.614	CV: 10	0.6405	CV: 8	0.6383	CV: 8	0.6515	CV: 10	0.639
	Precision	0.6198		0.6449		0.6421		0.6549		0.6443
	Recall	0.614		0.6405		0.6383		0.6515		0.639
	F1-score	0.6169		0.6427		0.6402		0.6532		0.6416
Hybrid CNN-LSTM CV: 10	Accuracy	0.4706	CV: 5	0.4621	CV: 10	0.4828	CV: 5	0.4874	CV: 8	0.4935
	Precision	0.527		0.5173		0.5463		0.5642		0.5723
	Recall	0.4706		0.4621		0.4828		0.4874		0.4935
	F1-score	0.4972		0.4881		0.5126		0.5230		0.530

Among the MLP models, models 2 and 3 exhibit moderate performance, with model 2 reaching its peak accuracy of 0.5798 at 20 s (fold 8) and model 3 achieving 0.5703 at 20 s (fold 5). However, these models show lower recall and F1-score than MLP Model 1, suggesting that they do not generalize as effectively.

Recurrent models, such as LSTM and GRU, demonstrate varying performance across different folds. The LSTM model performs the best at 19 s (fold 8) with an accuracy of 0.6378, while the GRU model reaches its peak at 19 s (fold 8) with an accuracy of 0.6515. This suggests that the performance of recurrent models remains highly dependent on the data split.

### 6.3 Performance analysis on AEC features

Using the AEC features, MLP model 2 achieves the highest overall performance, with an accuracy of 95.7%, precision of 95.8%, recall of 95.7%, and an F1-score of 95.8%. These values demonstrate the model's exceptional ability to classify instances correctly, minimize false positives, and identify true positives. The F1-score, which harmonizes precision and recall, confirms that MLP model 2 maintains a balanced performance without overemphasizing one aspect at the expense of the other. Its ability to leverage the AEC feature set likely stems from its optimized architecture, enabling it to capture intricate interconnections between features.

MLP model 3 also performs well, with an accuracy of 94.1%, precision of 94.2%, recall of 94.1%, and an F1-score of 94.2%. While its performance is lower than MLP model 2, the model demonstrates a robust ability to process the AEC feature set. It effectively generalizes across the dataset. The slight difference in performance compared to MLP model 2 could be attributed to architectural variations that make it less optimal in extracting fine-grained patterns.

MLP model 1 achieves an accuracy of 87.9%, precision of 87.9%, recall of 87.9%, and an F1-score of 87.9%. While these values are not as high as those of MLP models 2 and 3, model 1 still demonstrates an acceptable classification performance. It reliably identifies patterns in the AEC feature set, although its simpler architecture may limit its performance.

The simple RNN model performs well, achieving an accuracy of 90.5%, precision of 90.7%, recall of 90.5%, and an F1-score of 90.6%. These results highlight simple RNN's ability to generalize effectively on the AEC feature set, despite its recurrent architecture designed primarily for sequential data. Though its performance surpasses that of MLP model 1, it does not match the performance of MLP models 2 and 3, indicating that it does not exploit the full potential of the AEC feature set as efficiently as the MLP architectures.

The LSTM model exhibits a performance similar to simple RNN, with an accuracy of 90.6%, precision of 90.8%, recall of 90.6%, and an F1-score of 90.7%. This shows that while LSTM is a powerful model for sequential data, its complexity may not provide substantial benefits when applied to static connectivity features like AEC.

The GRU model achieves an accuracy of 90.3%, precision of 90.4%, recall of 90.3%, and an F1-score of 90.3%. While these values are close to those of simple RNN and LSTM, the slightly lower values suggest that it may be less suited for AEC than the other recurrent models.

Table 7 presents the accuracy of different deep learning models for AEC across various window sizes with an 80-20 fixed train-test split. MLP Model 2 consistently achieves higher accuracy across different window sizes, with values above 95%, making it the better-performing model for AEC. Among recurrent models, simple RNN performs better than LSTM and GRU, with its best accuracy at 17 s.

**Table 7:** Accuracy with AEC features for different window sizes with 80-20 fixed train-test split.

**Model**	**16 s**	**17 s**	**18 s**	**19 s**	**20 s**
MLP model 1	0.8851	0.8864	0.8765	0.8838	0.8788
MLP model 2	0.9626	0.9546	0.9624	0.9561	0.9572
MLP model 3	0.9514	0.9486	0.952	0.943	0.9412
Simple RNN	0.9049	0.9126	0.909	0.9026	0.9047
LSTM	0.8535	0.8467	0.8388	0.8303	0.9063
GRU	0.8506	0.8599	0.8524	0.8503	0.9028
Hybrid CNN-LSTM	0.925	0.9293	0.9272	0.9242	0.9273

The group k-fold cross-validation results using AEC-based features shown in Table 8 indicate that MLP models achieve higher accuracy across all tested models, with MLP Models 2 and 3 performing better. MLP Model 2 records a high accuracy of 0.9648 at 20 s (fold 8), followed closely by MLP Model 3 at 0.9648 at 19 s (fold 10). These results demonstrate the robust performance of MLP models when leveraging AEC-based features, suggesting that these models effectively capture the distinct patterns in the data. MLP Model 1 also shows strong performance, achieving its highest accuracy of 0.8895 at 16 s (fold 8), but is outperformed by the other two MLP models.

**Table 8:** Best fold obtained using group k-fold cross validation and the performance matrix for each model using AEC.

**16 s**			**17 s**		**18 s**		**19 s**		**20 s**	
MLP model 1 CV: 8	Accuracy	0.8895	CV: 8	0.8885	CV: 8	0.8839	CV: 8	0.8876	CV: 8	0.8834
	Precision	0.8933		0.8916		0.8883		0.891		0.8869
	Recall	0.8895		0.8885		0.8839		0.8876		0.8834
	F1-score	0.8914		0.8900		0.8861		0.8893		0.8851
MLP model 2 CV: 10	Accuracy	0.964	CV: 8	0.9644	CV: 8	0.9636	CV: 8	0.9642	CV: 8	0.9648
	Precision	0.9645		0.9651		0.9644		0.9648		0.9654
	Recall	0.964		0.9644		0.9636		0.9642		0.9648
	F1-score	0.9642		0.9647		0.9640		0.9645		0.9651
MLP model 3 CV: 10	Accuracy	0.9639	CV: 10	0.9639	CV: 8	0.964	CV: 8	0.9648	CV: 10	0.9622
	Precision	0.9644		0.9645		0.9644		0.9653		0.9631
	Recall	0.9639		0.9639		0.964		0.9648		0.9622
	F1-score	0.9641		0.9642		0.9642		0.9650		0.9626
Simple RNN CV: 10	Accuracy	0.916	CV: 5	0.9192	CV: 8	0.9225	CV: 5	0.9189	CV: 10	0.9212
	Precision	0.9169		0.9197		0.9233		0.9198		0.9223
	Recall	0.916		0.9192		0.9225		0.9189		0.9212
	F1-score	0.9164		0.9194		0.9229		0.9193		0.9217
LSTM CV: 8	Accuracy	0.8576	CV: 8	0.8595	CV: 8	0.8567	CV: 10	0.8564	CV: 10	0.8587
	Precision	0.8584		0.8602		0.8575		0.8573		0.8594
	Recall	0.8576		0.8595		0.8567		0.8564		0.8587
	F1-score	0.8580		0.8598		0.8571		0.8568		0.8590
GRU CV: 8	Accuracy	0.8641	CV: 10	0.8645	CV: 5	0.8686	CV: 5	0.8627	CV: 8	0.8705
	Precision	0.8649		0.8651		0.8694		0.863		0.8716
	Recall	0.8641		0.8645		0.8686		0.8627		0.8705
	F1-score	0.8645		0.8648		0.8690		0.8628		0.8710
Hybrid CNN-LSTM CV: 10	Accuracy	0.9283	CV: 8	0.933	CV: 5	0.9324	CV: 5	0.9293	CV: 10	0.9315
	Precision	0.9292		0.9336		0.933		0.9304		0.9321
	Recall	0.9283		0.933		0.9324		0.9293		0.9315
	F1-score	0.9287		0.9332		0.9326		0.9297		0.9317

Among RNN-based models, simple RNN exhibits the best performance, achieving an accuracy of 0.9225 at 18 s (fold 5), indicating that it effectively leverages AEC features. GRU and LSTM follow, with GRU reaching its highest accuracy of 0.8705 at 20 s (fold 8), while LSTM peaks at 0.8595 at 17 s (fold 8). These results highlight that RNN-based models, particularly simple RNN, still perform well with AEC-based features, though they did not surpass the MLP models in accuracy.

### 6.4 Performance analysis on combined features

MLP model 2 performs the best with accuracy, precision, recall, and F1-score values of 95.1%, 95.2%, 95.1%, and 95.1%, respectively. The high accuracy value indicates that the model reliably distinguishes between the classes, while the closely aligned precision and recall values reflect its ability to minimize false positives and false negatives effectively. However, these values are marginally lower than the values achieved by using AEC alone as the feature.

MLP model 3 performs slightly poorer than model 2. Model 3 achieves identical values of 94.8% for accuracy, precision, recall, and F1-score. The model's architecture appears well-suited for capturing the intricate relationships between wPLI and AEC features.

MLP model 1, with an accuracy of 87.4%, precision of 87.6%, recall of 87.4%, and an F1-score of 87.5%, exhibits a noticeable drop in performance compared to the other two MLP models. These values suggest that while MLP model 1 can handle the combined wPLI and AEC feature set, its simpler architecture might limit its capacity to fully capture the relationships between the two types of connectivity information.

Among the recurrent models, GRU achieves the highest performance, with equal values of accuracy, precision, recall, and F1-score of 92.1%. GRU's architecture captures the patterns of the combined wPLI-AEC features. LSTM performs poorer, with equal values of accuracy, precision, recall, and F1-score of 91.8%. The simple RNN model, while achieving an accuracy of 90.2%, precision of 90.4%, recall of 90.2%, and an F1-score of 90.3%, performs moderately well but falls short of the other recurrent and MLP architectures.

Table 9 displays the accuracy of various models utilizing AEC+wPLI for different window sizes (16-20 s) under an 80-20 train-test split. MLP-based models outperform recurrent models, with MLP model 2 consistently achieving better accuracy across all window sizes. Simple RNN performs better than LSTM and GRU in most cases, though GRU shows the highest accuracy of 0.9209 at 20 s, surpassing even LSTM. The best performance occurs at 18 s for MLP and simple RNN, while LSTM and GRU peak at 20 s.

**Table 9:** Accuracy with AEC+wPLI features for different window sizes with 80-20 train test split.

**Model**	**16 s**	**17 s**	**18 s**	**19 s**	**20 s**
MLP model 1	0.9083	0.8972	0.9167	0.9042	0.8735
MLP model 2	0.9685	0.9686	0.955	0.9639	0.9511
MLP model 3	0.9636	0.9671	0.9587	0.9584	0.9475
Simple RNN	0.9143	0.9099	0.9257	0.9076	0.9019
LSTM	0.8697	0.87	0.8564	0.8735	0.9177
GRU	0.8708	0.8771	0.8402	0.8756	0.9209
Hybrid CNN-LSTM	0.9015	0.915	0.9175	0.9155	0.925

Table 10 shows that the best fold obtained using group k-fold cross-validation across different models utilizing a combination of AEC and wPLI features varies based on the evaluation metrics. Among the models, MLP model 1 shows overall higher performance, achieving an accuracy of 0.9453 in its best fold (CV: 10). It also achieves a better precision (0.9463), recall (0.9453), and F1-score (0.9458). MLP Model 2 followed closely, with a best accuracy of 0.9383 (CV: 8), while MLP Model 3 has a slightly lower peak accuracy of 0.928 (CV: 8). Among recurrent models, GRU outperforms simple RNN and LSTM, achieving a better accuracy of 0.8581 (CV: 10), along with an F1-score of 0.8597, making it the strongest RNN-based model. The simple RNN and LSTM models display relatively lower accuracy, with the simple RNN peaking at 0.7518 (CV: 5) and LSTM at 0.756 (CV: 8), indicating that MLP models perform significantly better in this setup. Smaller window sizes (16-18 s) tend to yield better performance, especially for MLP models, which consistently achieve higher accuracy in this range. GRU models perform reliably across different window sizes but achieve their high results with 16 s, indicating that this window provides better informative features. Recurrent models (RNN and LSTM) exhibit some variability, with LSTM performing best at 16 s, while simple RNN shows slightly better results at 17 and 20 s.

**Table 10:** Best performing fold in group k-fold cross validation and the performance metrics for each model using combined AEC and wPLI features.

**16 s**			**17 s**		**18 s**		**19 s**		**20 s**	
MLP model 1 CV: 10	Accuracy	0.9453	CV: 10	0.9107	CV: 10	0.9437	CV: 10	0.9434	CV: 8	0.9118
	Precision	0.9463		0.9136		0.9456		0.9449		0.9133
	Recall	0.9453		0.9107		0.9437		0.9434		0.9118
	F1-score	0.9458		0.9121		0.9447		0.9441		0.9125
MLP model 2 CV: 10	Accuracy	0.9324	CV: 10	0.9051	CV: 10	0.9184	CV: 10	0.9336	CV: 8	0.9383
	Precision	0.9345		0.9087		0.9212		0.9349		0.9391
	Recall	0.9324		0.9051		0.9184		0.9336		0.9383
	F1-score	0.9334		0.9069		0.9198		0.9342		0.9387
MLP model 3 CV: 10	Accuracy	0.9256	CV: 8	0.9076	CV: 10	0.9172	CV: 8	0.928	CV: 5	0.9109
	Precision	0.9273		0.9105		0.9199		0.9292		0.9144
	Recall	0.9256		0.9076		0.9172		0.928		0.9109
	F1-score	0.9264		0.9090		0.9185		0.9286		0.9126
Simple RNN *CV*:10	Accuracy	0.7331	CV: 5	0.7518	CV: 10	0.7147	CV: 8	0.7364	CV: 8	0.7496
	Precision	0.7403		0.7555		0.72		0.7396		0.7556
	Recall	0.7331		0.7518		0.7147		0.7364		0.7496
	F1-score	0.7367		0.7536		0.7173		0.7380		0.7526
LSTM CV: 8	Accuracy	0.756	CV: 10	0.7339	CV: 10	0.7122	CV: 5	0.7137	CV: 8	0.7447
	Precision	0.7616		0.7374		0.7152		0.7212		0.7497
	Recall	0.756		0.7339		0.7122		0.7137		0.7447
	F1-score	0.7588		0.7356		0.7137		0.7174		0.7472
GRU *CV*:10	Accuracy	0.8581	CV: 10	0.8499	CV: 5	0.855	CV: 8	0.8576	CV: 10	0.8449
	Precision	0.8613		0.8537		0.8555		0.858		0.8465
	Recall	0.8581		0.8499		0.855		0.8576		0.8449
	F1-score	0.8597		0.8518		0.8552		0.8578		0.8457
Hybrid CNN-LSTM CV: 5	Accuracy	0.9047	CV: 10	0.9243	CV: 10	0.9189	CV: 5	0.9166	CV: 10	0.9266
	Precision	0.908		0.9251		0.9214		0.9189		0.9277
	Recall	0.9047		0.9243		0.9189		0.9166		0.9266
	F1-score	0.9063		0.9247		0.9201		0.9177		0.9271

The 95% confidence intervals are calculated for accuracy, precision, recall, and F1-score to evaluate the performance of different models using the four feature sets, namely PLI, wPLI, AEC, and combined AEC and wPLI. Among the models, MLP models 2 and 3 demonstrate higher accuracy, particularly with AEC (0.964, 95% CI = 0.964–0.965), while MLP model 1 performs slightly lower but improves significantly when using the combined AEC and wPLI (0.931, 95% CI = 0.909–0.953). The recurrent models (Simple RNN, LSTM, and GRU) show varying results, with GRU outperforming LSTM and RNN, particularly when using AEC and wPLI combinedly (0.853, 95% CI = 0.846–0.86). The CNN-LSTM hybrid model achieves better performance, attaining 0.931 accuracy (95% CI = 0.928–0.933) with AEC and 0.918 (95% CI = 0.908–0.929) with AEC and wPLI combined, making it comparable to the better-performing MLP models. Across all models, AEC consistently yields the highest performance, while the combination of AEC with wPLI further improves the results, particularly for MLP and GRU models. Table 11 presents the detailed confidence intervals for the implemented models.

**Table 11:** Confidence interval for the implemented models using different features.

**Model**	**Performance metrics**	**PLI**	**wPLI**	**AEC**	**AEC+wPLI**
MLP model 1	Accuracy	0.567 (0.555, 0.578)	0.776 (0.764, 0.787)	0.887 (0.883, 0.89)	0.931 (0.909, 0.953)
	Precision	0.571 (0.559, 0.582)	0.777 (0.765, 0.788)	0.89 (0.887, 0.893)	0.933 (0.911, 0.955)
	Recall	0.567 (0.555, 0.578)	0.776 (0.764, 0.787)	0.887 (0.883, 0.89)	0.931 (0.909, 0.953)
	F1-score	0.569 (0.557, 0.58)	0.776 (0.765, 0.788)	0.888 (0.885, 0.892)	0.932 (0.91, 0.954)
MLP model 2	Accuracy	0.628 (0.617, 0.64)	0.558 (0.54, 0.576)	0.964 (0.964, 0.965)	0.926 (0.909, 0.942)
	Precision	0.629 (0.617, 0.641)	0.662 (0.647, 0.676)	0.965 (0.964, 0.965)	0.928 (0.912, 0.943)
	Recall	0.628 (0.617, 0.64)	0.558 (0.54, 0.576)	0.964 (0.964, 0.965)	0.926 (0.909, 0.942)
	F1-score	0.661 (0.649, 0.673)	0.605 (0.59, 0.62)	0.964 (0.964, 0.965)	0.927 (0.91, 0.943)
MLP model 3	Accuracy	0.629 (0.616, 0.641)	0.56 (0.544, 0.575)	0.964 (0.963, 0.965)	0.918 (0.907, 0.929)
	Precision	0.63 (0.617, 0.642)	0.655 (0.641, 0.669)	0.964 (0.963, 0.965)	0.92 (0.91, 0.93)
	Recall	0.629 (0.616, 0.641)	0.56 (0.544, 0.575)	0.964 (0.963, 0.965)	0.918 (0.907, 0.929)
	F1-score	0.63 (0.617, 0.643)	0.604 (0.592, 0.615)	0.964 (0.963, 0.965)	0.919 (0.909, 0.93)
Simple RNN	Accuracy	0.529 (0.52, 0.538)	0.702 (0.683, 0.721)	0.92 (0.916, 0.923)	0.737 (0.719, 0.756)
	Precision	0.536 (0.526, 0.547)	0.704 (0.686, 0.723)	0.92 (0.917, 0.924)	0.742 (0.724, 0.76)
	Recall	0.529 (0.52, 0.538)	0.702 (0.683, 0.721)	0.92 (0.916, 0.923)	0.737 (0.719, 0.756)
	F1-score	0.487 (0.478, 0.497)	0.703 (0.685, 0.722)	0.92 (0.917, 0.923)	0.74 (0.721, 0.758)
LSTM	Accuracy	0.487 (0.482, 0.493)	0.628 (0.616, 0.639)	0.858 (0.856, 0.859)	0.732 (0.708, 0.756)
	Precision	0.492 (0.486, 0.498)	0.631 (0.619, 0.643)	0.859 (0.857, 0.86)	0.737 (0.713, 0.761)
	Recall	0.487 (0.482, 0.493)	0.628 (0.616, 0.639)	0.858 (0.856, 0.859)	0.732 (0.708, 0.756)
	F1-score	0.469 (0.462, 0.477)	0.629 (0.617, 0.641)	0.858 (0.857, 0.86)	0.735 (0.711, 0.758)
GRU	Accuracy	0.501 (0.495, 0.506)	0.637 (0.62, 0.654)	0.866 (0.862, 0.87)	0.853 (0.846, 0.86)
	Precision	0.506 (0.501, 0.511)	0.641 (0.625, 0.657)	0.867 (0.862, 0.871)	0.855 (0.848, 0.862)
	Recall	0.501 (0.495, 0.506)	0.637 (0.62, 0.654)	0.866 (0.862, 0.87)	0.853 (0.846, 0.86)
	F1-score	0.51 (0.502, 0.519)	0.639 (0.622, 0.655)	0.866 (0.862, 0.871)	0.854 (0.847, 0.861)
Hybrid CNN-LSTM	Accuracy	0.573 (0.563, 0.582)	0.479 (0.463, 0.495)	0.931 (0.928, 0.933)	0.918 (0.908, 0.929)
	Precision	0.574 (0.564, 0.583)	0.545 (0.516, 0.575)	0.932 (0.929, 0.934)	0.92 (0.911, 0.93)
	Recall	0.573 (0.563, 0.582)	0.479 (0.463, 0.495)	0.931 (0.928, 0.933)	0.918 (0.908, 0.929)
	F1-score	0.573 (0.564, 0.582)	0.51 (0.489, 0.532)	0.931 (0.929, 0.934)	0.919 (0.909, 0.929)

### 6.5 Sliding window correlation analysis

The sliding window correlation analysis of weighted phase-lag index and amplitude envelope correlation connectivity matrices across four groups: coma, UWS, MCS, and healthy controls provides critical insights into the brain's functional connectivity and its relationship to levels of consciousness. By examining the variability of connectivity patterns across five frequency bands (delta, theta, alpha, beta, gamma), the study captures the nature of neural interactions that underpin different conscious states. Two key metrics, mean SWC and standard deviation (Std. SWC), are used to quantify these patterns. The mean SWC reflects the overall similarity of connectivity across consecutive windows, while Std. SWC captures the variability of these patterns over time, offering a detailed view of the network dynamics.

#### 6.5.1 SWC analysis for PLI

##### 6.5.1.1 Group-level interpretation of mean SWC

In the delta band, mean SWC values of PLI rise steadily from coma (0.0196) to healthy controls (0.0593), reflecting enhanced functional connectivity as the brain transitions to more conscious states. This pattern is consistent across other frequency bands, such as theta (0.0208 in coma to 0.0540 in healthy controls), alpha (0.0222–0.0602), beta (0.0363–0.0554), and gamma (0.0183–0.0586). These increases suggest that higher levels of consciousness are associated with stronger interactions within brain networks. Among the groups, healthy controls consistently exhibit the highest mean SWC values across all frequency bands, indicating robust connectivity in fully conscious individuals. Conversely, coma patients display the lowest values, signifying diminished network interactions. UWS and MCS groups generally fall between these extremes, with subtle differences observed particularly in theta and beta bands.

#### 6.5.2 Variability in connectivity

The standard deviation provides a measure of variability in connectivity across groups and reveals distinct trends. Healthy controls consistently show the highest variability across all frequency bands, with Std SWC values peaking in the delta (0.1446), theta (0.1428), alpha (0.1431), beta (0.1381), and gamma (0.1447) bands. This variability underscores the complexity and adaptability of brain network interactions in fully conscious individuals. In contrast, coma patients exhibit lower variability, indicating more uniform connectivity values. UWS and MCS groups show intermediate variabilities, with overlapping patterns in theta and gamma bands. This suggests that while both states share similarities in their connectivity, the greater heterogeneity in MCS may reflect the emergence of more complex neural interactions.

#### 6.5.3 SWC analysis for wPLI

##### 6.5.3.1 Group-level interpretation of mean SWC

Across all frequency bands, healthy controls consistently exhibit the highest mean SWC values, highlighting robust connectivity. In the delta band, HC have a mean SWC of 0.034, while coma patients have only 0.00003, showing diminished connectivity. Similarly, in the theta band, mean SWC is progressively higher across the groups: coma (0.0038), UWS (0.0061), MCS (0.029), and HC (0.054), reflecting a gradual recovery of stable network with improving consciousness levels. This trend is consistent across the alpha band, where mean SWC ranges from -0.0025 in coma to 0.035 in HC, and the beta band, where values range from -0.0067 in coma to 0.034 in HC. The gamma band, associated with high-frequency neural processes, shows a slightly different pattern. While HC has a high mean SWC of 0.030, the MCS group exhibits the highest mean SWC of 0.036, potentially indicating a unique pattern of gamma connectivity during partial recovery of consciousness. On the other hand, coma patients show reduced mean SWC values in the gamma band (0.0075), revealing a lack of high-frequency connectivity in them.

#### 6.5.4 Variability in connectivity

High variability is often interpreted as an indicator of flexible and adaptive neural networks, which are hallmarks of healthy brain function. Here, healthy controls demonstrate significantly greater Std. SWC values across most frequency bands. In the theta band, HC show a Std. SWC of 0.236, compared to 0.178 in coma and 0.200 in UWS. Also in the alpha band, where HC have the highest Std. SWC (0.243), indicating network flexibility, coma patients exhibit the lowest Std. SWC (0.178). A similar pattern is seen in the beta band, with Std. SWC values increasing progressively from coma (0.178) to HC (0.241). In the gamma band, HC again displays the highest Std. SWC (0.244); MCS follows closely with 0.188, potentially reflecting a recovery in high-frequency network flexibility as consciousness is partially restored. Lower Std. SWC in coma patients (0.175) suggests that their FC networks are less capable of adapting, which is consistent with the rigidity observed in severely impaired states.

### 6.5.5 SWC analysis for AEC

#### 6.5.5.1 Group-level interpretation of mean SWC

Healthy controls exhibit the highest mean SWC values across all frequency bands, underscoring their robust connectivity. HC show a mean SWC of 0.6487, significantly higher than coma (0.3093) and MCS (0.3098) in delta band. This indicates that low-frequency connectivity in healthy brains is consistently more stable than those in pathological states, reflecting a well-integrated and synchronized network. UWS patients, with a mean SWC of 0.4406 in the delta band, occupy an intermediate position, suggesting partial recovery of network compared to coma. In the theta band, HC maintains the highest mean SWC (0.6609), compared to coma (0.3458), UWS (0.4119), and MCS (0.3271). Theta oscillations are critical for memory and attention, and the marked difference in mean SWC between HC and pathological groups highlights their diminished capacity for cognitive processes. The alpha band, often associated with sensory processing and resting-state connectivity, shows a similar progression. HC exhibit a mean SWC of 0.6409, more than double that of coma (0.3072). UWS and MCS show slightly closer values (0.3681 and 0.3267, respectively), indicating some preservation of alpha connectivity in these groups, albeit significantly disrupted compared to healthy individuals. In the beta band, which supports higher-order cognitive functions, HC has the highest connectivity, with a mean SWC of 0.6591, compared to 0.3214 for coma, 0.3962 for UWS, and 0.3264 for MCS. In the gamma band, critical for neural synchronization and cognitive integration, HC (mean SWC = 0.6490) exhibit far more connectivity than coma (0.3445) and MCS (0.3395).

### 6.5.6 Variability in connectivity

Healthy controls exhibit moderate variability in most bands, with Std. SWC values consistently lower than mean SWC, reflecting an optimal balance between stability and flexibility. For instance, in the delta band, HC have a Std. SWC of 0.1984, significantly lower than their mean SWC of 0.6487, suggesting that low-frequency networks in healthy individuals are highly stable with low fluctuation. Coma and MCS groups exhibit slightly higher variability in the delta band (0.2526 and 0.2506, respectively), likely reflecting unstable network. UWS patients, with a Std. SWC of 0.2396, exhibit intermediate variability, consistent with their partial preservation of flexibility. In the theta band, HC show a Std. SWC of 0.2064, again reflecting a stable yet adaptable network. Comparatively, coma (0.2444) and MCS (0.2617) show higher variability, possibly indicating disorganized connectivity patterns. The UWS group exhibits variability comparable to HC (0.2467), suggesting that theta-band networks retain some flexibility in this group. The alpha band provides a clearer differentiation between groups. HC, with a Std. SWC of 0.2120, demonstrate the lowest variability, consistent with well-regulated alpha connectivity. In contrast, coma and MCS groups have significantly higher variability (0.2616 and 0.2560, respectively), indicating erratic alpha connectivity. UWS patients again occupy an intermediate position, with a Std. SWC of 0.2519, suggesting partial preservation of alpha flexibility. In the beta band, HC exhibit the lowest Std. SWC (0.1952), indicating that their high-frequency FC is both stable and efficient. Pathological groups show higher variability, with Std. SWC values of 0.2378 (coma), 0.2664 (UWS), and 0.2616 (MCS). The gamma band follows a similar trend, with HC showing a Std. SWC of 0.2266, while coma, UWS, and MCS have values of 0.2394, 0.2579, and 0.2541, respectively.

## 7 Discussion

The study analyses the classification ability of functional connectivity features, namely PLI, wPLI, and AEC, using various artificial neural network models. Across all feature sets, MLP model 2 consistently outperforms others in terms of accuracy, precision, recall, and F1-score. With the wPLI feature, MLP model 2 achieves an accuracy of 0.772. With the AEC feature, it delivers higher performance, achieving an accuracy of 0.963. When combining wPLI and AEC features, MLP model 2 gives a marginally higher accuracy of 0.969.

Whereas, PLI feature performs poorly across all models and metrics, consistently showing results inferior to other features. On MLP model 1, it achieves an accuracy of only 0.559, which is markedly lower than AEC (0.886) and wPLI (0.772). Its performance remains poor in recurrent models like LSTM and GRU, where it records accuracies of just 0.533 and 0.529, respectively. Similarly, its F1-scores are weak, hovering around 0.544. These results indicate that PLI is less effective in capturing connectivity patterns than wPLI and AEC in all evaluations.

These results highlight that MLP model 2 is the best-performing model for this classification task, with the AEC feature providing the best result across all performance metrics. This emphasizes the strength of amplitude-based connectivity metrics for distinguishing between consciousness states.

Table 12 presents a detailed comparison of our results with the techniques in the literature for distinguishing states of consciousness, including coma, UWS, MCS, and HC. Among existing methods for classifying disorders of consciousness, Naro et al. (2018) focused on phase-based connectivity and analyzed debiased weighted phase lag index (dwPLI) feature in 15 MCI and 17 UWS subjects. However, the absence of key performance metrics like accuracy and precision limits the comparability of their findings. Similarly, Di Gregorio et al. (2022) employed dominant frequency and phase coherence features with a linear discriminant analysis classifier to classify DOC subjects based on traumatic brain injury status. The study achieved accuracies of 80% and 83.3% for TBI and Non-TBI groups, with precision values of 0.777 and 0.857. While these results demonstrate the utility of simple classifiers, reliance on linear methods constrains their ability to capture complex relationships characteristic of brain waves.

**Table 12:** Comparison of our performance with the techniques in the literature.

**References**	**Features**	**No. of subjects**	**Classifier**	**Accuracy (%)**	**Precision (%)**
Naro et al. (2018)	dwPLI	MCS 15, UWS 17	-	-	-
Di Gregorio et al. (2022)	Dominant frequency, PCoh, MI	DOC 33	LDA	TBI: 80; Non-TBI: 83.3	77.7; 85.7
Duclos et al. (2021)	wPLI, AEC	Controls 9 (anesthetic-induced unconsciousness)	SVM	AEC: 83.7; wPLI: 69.4; AEC + wPLI: 84.1	-
Li et al. (2024a,b)	Microstate, dFC: MI	MCS: 16 UWS: 16	CatBoost	96.2	-
Raveendran et al. (2024)	VMD mode-based features: kurtosis, skewness, spectral entropy, sample entropy	Coma 15, UWS 15, MCS 15	Ensemble bagged tree	UWS vs. MCS: 83.3 Coma vs. UWS vs. MCS:76.0	84.278.9
Proposed method	FC feature: AEC	Coma 15, UWS 15, MCS 15, controls 15	ANN models	96.3	96.3

TBI, traumatic brain injury.

The study by Duclos et al. (2021) explored the application of FC measures like wPLI and AEC to assess anesthetic-induced unconsciousness and classify various altered consciousness states. SVM classifier was employed to analyze the anesthetic-induced unconsciousness in nine control subjects, reporting accuracies of 69.4% for wPLI, 83.7% for AEC, and 84.1% for their combination. However, the study included only nine subjects. Ensemble approaches, such as the CatBoost classifier used by Li et al. (2024a) and the bagged tree model in Raveendran et al. (2024), demonstrate improved adaptability but may lack the flexibility of ANN models when processing diverse and complex feature sets.

The inclusion of both accuracy and precision metrics strengthens the proposed method's evaluation. Accuracy measures the overall correctness of classification, while precision quantifies the model's ability to avoid false positives. This dual assessment addresses a notable gap in existing studies, where a sole focus on accuracy may obscure the clinical relevance of results. By providing a balanced evaluation, the proposed method ensures reliability and effectiveness, critical for clinical applications where misclassification can have significant consequences.

Our findings support integrating FC features, since combining wPLI and AEC features marginally improves the classification performance.

### 7.1 Limitations and future directions

While this study provides valuable insights, several limitations must be acknowledged. Firstly, the study relies on training and testing data from a fixed set of participants, which limits the generalizability of the findings. Despite demonstrating better performance metrics, the models' applicability to new datasets or broader population remains uncertain. Further, the analysis is restricted to three functional connectivity features: PLI, wPLI, and AEC. The study assumes that input features are standardized and preprocessed consistently across all states of consciousness. While this approach ensures comparability, it may overlook subtle variations in feature distributions across classes. To mitigate these limitations, future research will consider integrating multiple connectivity features to enhance classification performance.

#### 7.1.1 Implications for clinical applications

The ability to accurately classify states of consciousness has significant clinical implications for the diagnosis and management of DOC. The study's findings suggest that MLP architectures using AEC as feature offer a reliable and practical solution for clinical applications. The high accuracy and balanced performance values achieved by these models indicate their potential for automated diagnostic systems. For applications focusing on phase-based features, GRU models leveraging wPLI features provide a complementary approach. By combining both approaches, a multimodal framework can be developed that integrates amplitude and phase-based connectivity measures to improve diagnostic accuracy and patient outcome.

## 8 Conclusion

This study demonstrates the importance of aligning feature selection and model design in classifying states of consciousness. AEC's superior predictive power highlights its utility as a standalone feature, while wPLI quantifies the phase relationship between channels. The findings emphasize the complementary nature of these features and the necessity of tailoring ANN architectures to their unique characteristics. By leveraging these insights, researchers can develop more effective tools for diagnosing and understanding disorders of consciousness, paving the way for improved patient care and deeper insights into the neural mechanisms underlying consciousness. Future work can explore other features, advanced architectures, and independent datasets to further refine these approaches and enhance their clinical utility.

## Data Availability

The raw data supporting the conclusions of this article will be made available by the authors, without undue reservation.
